# Mobile Apps to Support Mental Health Response in Natural Disasters: Scoping Review

**DOI:** 10.2196/49929

**Published:** 2024-04-17

**Authors:** Nwamaka Alexandra Ezeonu, Attila J Hertelendy, Medard Kofi Adu, Janice Y Kung, Ijeoma Uchenna Itanyi, Raquel da Luz Dias, Belinda Agyapong, Petra Hertelendy, Francis Ohanyido, Vincent Israel Opoku Agyapong, Ejemai Eboreime

**Affiliations:** 1 Center for Translation and Implementation Research College of Medicine University of Nigeria Nsukka Nigeria; 2 Department of Information Systems and Business Analytics College of Business Florida International University Miami, FL United States; 3 Department of Emergency Medicine Beth Israel Deaconess Medical Center and Harvard Medical School Boston, MA United States; 4 Department of Psychiatry Faculty of Medicine Dalhousie University Halifax, NS Canada; 5 Geoffrey and Robyn Sperber Health Sciences Library University of Alberta Edmonton, AB Canada; 6 Department of Community Medicine University of Nigeria Enugu Nigeria; 7 Department of Public Health Sciences Dalla Lana School of Public Health University of Toronto Toronto, ON Canada; 8 Department of Psychiatry Faculty of Medicine and Dentistry University of Alberta Edmonton, AB Canada; 9 Department of Psychology Florida State University Tallahassee, FL United States; 10 West African Institute of Public Health Abuja Nigeria

**Keywords:** mental health, disasters, mobile health, mHealth, application, applications, app, apps, smartphone, stress, psychological, traumatic, disaster, disasters, hazard, hazards, emergency, psychological trauma, mobile apps, trauma, scoping, review methods, review methodology, mobile phone

## Abstract

**Background:**

Disasters are becoming more frequent due to the impact of extreme weather events attributed to climate change, causing loss of lives, property, and psychological trauma. Mental health response to disasters emphasizes prevention and mitigation, and mobile health (mHealth) apps have been used for mental health promotion and treatment. However, little is known about their use in the mental health components of disaster management.

**Objective:**

This scoping review was conducted to explore the use of mobile phone apps for mental health responses to natural disasters and to identify gaps in the literature.

**Methods:**

We identified relevant keywords and subject headings and conducted comprehensive searches in 6 electronic databases. Studies in which participants were exposed to a man-made disaster were included if the sample also included some participants exposed to a natural hazard. Only full-text studies published in English were included. The initial titles and abstracts of the unique papers were screened by 2 independent review authors. Full texts of the selected papers that met the inclusion criteria were reviewed by the 2 independent reviewers. Data were extracted from each selected full-text paper and synthesized using a narrative approach based on the outcome measures, duration, frequency of use of the mobile phone apps, and the outcomes. This scoping review was reported according to the PRISMA-ScR (Preferred Reporting Items for Systematic Reviews and Meta-Analyses extension for Scoping Reviews).

**Results:**

Of the 1398 papers retrieved, 5 were included in this review. A total of 3 studies were conducted on participants exposed to psychological stress following a disaster while 2 were for disaster relief workers. The mobile phone apps for the interventions included Training for Life Skills, Sonoma Rises, Headspace, Psychological First Aid, and Substance Abuse and Mental Health Services Administration (SAMHSA) Behavioural Health Disaster Response Apps. The different studies assessed the effectiveness or efficacy of the mobile app, feasibility, acceptability, and characteristics of app use or predictors of use. Different measures were used to assess the effectiveness of the apps’ use as either the primary or secondary outcome.

**Conclusions:**

A limited number of studies are exploring the use of mobile phone apps for mental health responses to disasters. The 5 studies included in this review showed promising results. Mobile apps have the potential to provide effective mental health support before, during, and after disasters. However, further research is needed to explore the potential of mobile phone apps in mental health responses to all hazards.

## Introduction

Rising global average temperatures and associated changes in weather patterns result in extreme weather events that include hazards such as heatwaves, wildfires, hurricanes, floods, and droughts [1]. These extreme events linked to climate change are resulting in overlapping and so-called cascading disasters leading to record numbers of “billion dollar” disasters with significant losses of lives and property [2,3]. In 2021 alone, approximately 10,000 fatalities caused by disasters were reported globally, while the economic loss was estimated at approximately US $343 billion [4]. Disasters are predicted to become more recurring as a result of the impact of human activities such as burning fossil fuels and deforestation, which release greenhouse gases into the atmosphere that trap heat and cause global temperatures to rise [5].

These catastrophes can adversely affect physical health, mental health, and well-being in both the short and long term as a result of changes due to the political and socioeconomic content, evacuations, social disruption, damage to health care facilities, and financial losses [6-10]. It is estimated that about 33% of people directly exposed to natural disasters will experience mental health sequelae such as posttraumatic stress disorders (PTSDs), anxiety, and depression, among others [11,12].

There is growing recognition of the importance of incorporating mental health into medical and emergency aspects of disaster response [12,13]. However, in contrast to most medical response strategies that are largely curative, mental health response to disasters is predicated on the principles of preventive medicine, thus, emphasizing health promotion, disaster prevention, preparedness, and mitigation [14]. The strategies of mental health response span across primary prevention (mitigating the risk of ill health before it develops), secondary prevention (early detection and intervention), and tertiary prevention (managing established ailment and averting further complications) [15].

Mobile health (mHealth) technology has shown great promise in mental health and has been applied across the 3 levels of prevention [16-20]. For example, SMS text messaging and mobile apps have been developed to promote mental health awareness among young people and older adults (primary prevention) [21]. Additionally, during the COVID-19 pandemic, mHealth was deployed at the population level in Canada to screen for symptoms of anxiety and depression (secondary prevention) [22]. In addition, mHealth interventions were deployed to support first responders and essential workers during the pandemic [23,24]. Further, the technology has been deployed for therapeutic purposes in patients diagnosed with mental health conditions while simultaneously providing support against complications such as suicidal ideation (tertiary prevention) [25].

Although videoconferencing and phone calls can be used for mental health conditions, mobile apps provide more mobility and accessibility, are interactive, more adaptable to users’ routines, and can be used repeatedly [26,27]. While numerous academic studies have been conducted on the app of mHealth in the preventive and curative management of mental health conditions in clinical, community, and public health settings, including epidemic response and control, little is known about the use of mobile apps in the mental health components of natural disaster management. This scoping review aims to fill this gap in the literature by mapping where and how mobile apps have been used as part of natural disaster mental health response strategies.

## Methods

### Overview

This scoping review was reported according to the PRISMA-ScR (Preferred Reporting Items for Systematic Reviews and Meta-Analyses extension for Scoping Reviews) [28]. The PRISMA-ScR checklist is available in Multimedia Appendix 1. The protocol was not registered.

### Search Strategy

A medical librarian (JYK) collaborated with the research team to identify relevant keywords and subject headings for the review, such as mHealth or m-health; mobile health or mobile applications; public health emergency, disaster, or catastrophe; and flood, earthquake, or hurricane. Equipped with this knowledge, the librarian developed and executed comprehensive searches in 6 electronic databases, including Ovid MEDLINE, Ovid Embase, APA PsycInfo, CINAHL, Scopus, and Web of Science Core Collection. The search was conducted on June 30, 2022, and was limited to the English language. The full search strategies are available in Multimedia Appendix 2.

### Inclusion and Exclusion Criteria

We included papers that applied mobile apps for mental health responses to disasters. Papers were included if the study participants were persons affected by a natural disaster (setting), the intervention included using a mobile phone app, and the outcome included the assessment of a mental health problem. Studies in which participants were exposed to a man-made disaster were included if the sample also included some participants exposed to a natural disaster. The mental health conditions included were stress, anxiety, depression, and PTSD. Only full-text studies published in English were included. Studies that did not include any intervention with a mobile app for mental health, those focused on videoconferencing or phone calls, and papers on protocols, trial registration, or review were excluded.

### Selection of Studies

The search identified papers that were retrieved from the databases. After removing duplicates, the initial titles and abstracts of the unique papers were screened by 2 independent review authors based on the inclusion criteria in a web-based tool called Covidence (Veritas Health Innovation Ltd) [29]. Full texts of the selected papers that met the inclusion criteria were reviewed by the 2 independent reviewers. The research team resolved disagreements through discussion. The bibliographies from the included studies were also reviewed to identify additional studies for inclusion.

### Data Extraction and Synthesis

Data from each selected full-text paper were extracted into a data extraction form developed by the research team. The data included the author and year of publication, country of study, study design, number of participants, type of natural disaster, name of the mobile app, duration of use of the app, outcome measures, and the study’s findings. These data were synthesized using a narrative approach based on the outcome measures, the duration, frequency of use of the mobile apps, and the outcomes.

## Results

### Search Results

**Figure 1:**
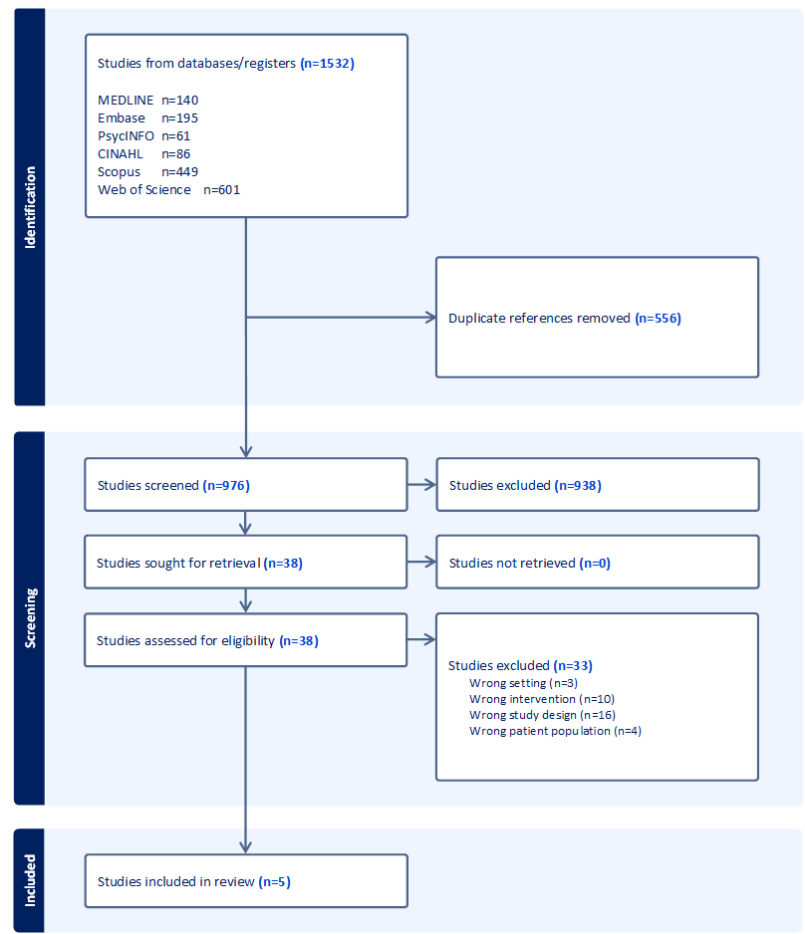
PRISMA diagram. PRISMA: Preferred Reporting Items for Systematic Reviews and Meta-Analyses.

Of the 1532 papers retrieved from the searches, 976 unique papers had their titles and abstracts screened after deduplication. A total of 38 papers were moved to full-text screening, and data were extracted from 5 papers [30-34] (Figure 1). Table 1 shows the summary of the details of the papers.

**Table 1:** Summary of studies using mobile phone apps for mental health response in disasters.

References	Country of study	Type of natural disaster	Mental health conditions targeted	Target population	Name of app (if available)	Intervention (app content)	Conclusion
Choi et al [32]	South Korea	Mixed: earthquakes—2 participants, fires—3 participants, hazardous chemical—4 participants, and traffic accidents—11 participants.	Post-traumatic stress, 1 year after a natural, social, or artificial disaster.	Disaster survivors	TLS^a^ apps	Information: self-assessment (PTSD^b^, depression, anxiety, and sleep disorder), postdisaster responses, coping mechanisms (anger, sleep disorder, and addiction) and guidance on how to use information from specialized institutions. Psychological healing: breathing, butterfly hug, meditation, positive affirmations, healing music, 108 bows, yoga, and writing. Ventilation and diversion (mood change category): find the same picture game, fruit slot machine, bubble shot, coloring book, and Tetris.	The TLS mobile app was effective for increasing positive and decreasing negative psychological factors according to app usage time and is expected to provide psychological stability and to provide the appropriate conditions to enable effective self-control and management for disaster survivors.
Heinz et al [30]	United States	Wildfires	PTSD and postdisaster distress	Disaster survivors	Sonoma Rises	The app includes 6 self-paced content sections, psychoeducation, and direct connections to free and local mental health care services. Participants completed daily ratings of anxiety and fear; weekly measures of post-traumatic stress symptoms; internalizing and externalizing symptoms; psychosocial functioning; and then measures of anxiety, depression, well-being, sleep, academic engagement, and perceived social support; and quantitative and qualitative measures of intervention satisfaction and feasibility.	Findings from this study suggest that adolescent disaster survivors are willing and able to use digital health tools and find them beneficial.
Rung et al [31]	United States	Hurricanes in 2005 and Deepwater Horizon oil spill in 2010	Stress prevention and management	Disaster survivors	The Headspace mobile mindfulness app	A standardized meditation program on MBSR^c^ used for at least 30 days, 10 minutes at a time.	The program was easy and cost-effective to implement and acceptable to those who participated, but few women elected to try it. Several short-term benefits of the program were identified, particularly for depression and sleep.
Choi et al [33]	South Korea	Disaster simulation training for responders in flood, fire, or leakage of hazardous chemicals	Traumatic stress	Disaster relief workers	PFA^d^ app	PFA preparation: self-assessment for relief worker, precautions for general and vulnerable group, precautions by disaster types. PFA implementation: forming therapeutic relationship; information gathering; and assessment of psychological status, skills for PFA, and linkage to community resources. Psychological healing: response after disaster relief, stress management, and institutions for support	The use of PFA mobile app during disaster simulation training can function as a new framework for providing disaster relief teaching and methodical mental health services to survivors at the disaster site by disaster health care staff.
Seligman et al [34]	United States	Any disaster	Stress prevention and management	Disaster relief workers	SAMHSA^e^ Behavioral Health Disaster Response App	The app provides access to trauma and disaster-related behavioral health resources available on the web or in hard copyright on a smartphone.	The SAMHSA disaster app assures that responders feel confident at having the best disaster behavior health resources.

^a^TLS: Training for Life Skills.

^b^PTSD: posttraumatic stress disorder.

^c^MBSR: Mindfulness-Based Stress Reduction.

^d^PFA: Psychological First Aid.

^e^SAMHSA: Substance Abuse and Mental Health Services Administration.

### Characteristics of Included Studies

Of the 5 studies included in this review, 3 (60%) were conducted in the United States [30,31,34], while 2 (40%) were conducted in South Korea [32,33]. All studies used different study designs. A total of 3 studies used a quasi-experimental design—the first, a single group postexperiment with 22 participants [32]; the second, a multiple-baseline single case experimental design with 7 participants [30], while the third study used a 1-group pre- and posttest design with 318 participants [31]. The Training for Life Skills (TLS) app study had only a posttest following the use of the app [32]; the other 2 had baseline and follow-up measurements with the Sonoma Rises app study having, in addition, preintervention and postintervention measurements. The Psychological First Aid (PFA) study was designed as a qualitative study, while the Substance Abuse and Mental Health Services Administration (SAMHSA) study used a mixed methods descriptive design.

### Characteristics of the Population

The TLS, Sonoma, and Headspace apps were designed for disaster survivors, while the PFA and SAMHA apps were designed to support disaster relief workers. The TLS app study was administered to adults with a median age of 32 years. Participants of the Sonoma Rises app study had a mean age of 16 (SD 0.98) years, while participants of the Headspace app study had a mean age of 46.1 (SD 10) years. The TLS app study focused on all types of disasters; the Sonoma Rises study focused on adolescents exposed to wildfires, while the Headspace app focused on women who experienced hurricanes and deep-water oil spillage. The PFA study involved 19 disaster health care workers who first underwent disaster simulation training using the mobile app.

### Characteristics of the Mobile App Interventions

The included studies revealed several mobile phone apps used as interventions. The first, the TLS app, was used as a psychological first aid program for disaster survivors with content on information, psychological healing, and mood change [32]. The second was the Sonoma Rises app, a Health Insurance Portability and Accountability Act (HIPAA)–compliant, cloud-based mobile app with daily push notifications as reminders designed to help survivors of wildfires or other disasters to find their new routines, build resilience, and increase well-being. The app included 6 self-paced content sections, psychoeducation, and direct connections to free and local mental health care services. The third was the Headspace app for a mindfulness-based stress reduction program that included a series consisting of 10 sessions designed to be used for about 10 minutes per day. The SAMHSA Disaster App equips behavioral health providers to respond to all kinds of traumatic incidents by enabling them to readily access disaster-specific information and other important materials directly on their mobile devices [34]. The PFA mobile app provided evidence-based information and tools for disaster workers to prepare for, execute, and recover from providing psychological first aid during disasters. Accessibility via smartphones and the inclusion of multimedia interventions and assessments tailored for disaster contexts were key features enabling its use integrated with the simulation training [33].

### Frequency and Duration of App Use

The 3 survivor-based apps had variations in the duration of the intervention (app use), which were 8 weeks, at least 5 times a week, frequency of use per day not specified [32]; 4 weeks for 10 minutes per day [30]; and 6 weeks for 5-10 minutes per day [31]. Both the TLS app and the Sonoma Rises app studies had weekly follow-up assessments. The different interventions were applied at least a year following the disasters. Participants in the Sonoma Rises app study used the app on an average of 17 (SD 8.92) days and visited the app an average of 43.50 (SD 30.56) times, with an average session lasting 56.85 (SD 27.87) seconds. The mean time spent on the app was 35.77 (SD 30.03) minutes, while for the TLS app study, the median time spent on the app over the 8 weeks of use was 200-399 minutes. Participants used the Headspace app an average of 24 (SD 36) days and logged in an average of 36 (SD 80) times. There was no description of the frequency and duration of use for the relief worker apps.

### Effectiveness Outcomes

Effectiveness outcomes refer to the effects or impact of an intervention or program on the intended outcomes or goals. Different measures were used to assess the effectiveness of the apps’ use as either the primary or secondary outcome. Emotional quotients (emotional stability), basic rhythm quotients (brain stability), alpha-blocking rates (increased positive mood), and brain quotients assessed using electroencephalogram (EEG)–measured brainwave activities adjusted for self-reported app use time were used in the TLS app study [32]. The Headspace app study assessed effectiveness using a combination of measures such as trait mindfulness using a 15-item Mindful Attention Awareness Scale (MAAS)—trait version; depressive symptoms using the Center for Epidemiologic Studies Depression Scale-10 (CESD-10); perceived stress with the Perceived Stress Scale, 4-item version (PSS-4); and sleep quality using the Pittsburgh Sleep Quality Index (PSQI) [31]. The Sonoma Rises app study measured efficacy using daily ratings of anxiety and fear, weekly measures of post-traumatic stress symptoms using the Child PTSD Symptom Scale (CPSS-5) for *Diagnostic and Statistical Manual of Mental Disorders, Fifth Edition* (*DSM-5*), internalizing and externalizing symptoms using the Behaviour and Feelings Survey (BFS), psychosocial functioning using the Ohio Scale for Youth—Functioning subscale (OSY), and measures of anxiety (Generalized Anxiety Disorder-7 [GAD-7]), depression (Patient Health Questionnaire-9 [PHQ-9]), well-being—Warwick-Edinburgh Mental Well-being Scale (WEMWBS), sleep (Insomnia—Severity Index [ISI]), academic engagement (Student Engagement Instrument [SEI]), and perceived social support (Wills’ Social Support Scale [WSSS]) [30].

All 3 survivor-based apps were found to have positive benefits in addressing mental health issues among persons exposed to natural disasters. The TLS mobile app was shown to be effective in increasing positive and decreasing negative psychological factors according to app use time. The TLS mobile apps’ use had a significant effect on the emotional quotients (β=.550; *P*<.008), explanatory power (EP) was 30%, had a significant positive effect on the basic rhythm quotient (left brain: β=.598; *P*<.003; EP 35; right brain: β=.451; *P*<.035; EP 20%). Additionally, it had a significant positive effect on the alpha-blocking rate (left brain: β=.510; *P*<.015; EP 26%; right brain: β=.463; *P*<.035, EP 21%); and a significant positive effect on the brain quotient (β=.451; *P*<.035; EP 20%) [16]. The Headspace app had a positive effect on depression (odds ratio [OR] 0.3, 95% CI 0.11-0.81), physical activity (OR 2.8, 95% CI 1.0-7.8), sleep latency (OR 0.3, 95% CI 0.11-0.81), sleep duration (OR 0.3, 95% CI 0.07-0.86), and sleep quality (OR 0.1, 95% CI 0.02-0.96); however, there was no change in mindfulness scores from baseline to follow-up. For the Sonoma Rises app, no significant effects were observed for the clinical and functional outcomes because the longitudinal part of the study was affected by limited statistical power as a result of small sample size and historical confounds that made the participants miss data submission. However, visual inspection of individual data following the intervention showed downward trends across the study phases for daily levels of anxiety, fearfulness, and individual posttraumatic stress symptom severity.

For the PFA app, the qualitative study explored disaster health workers’ experiences with simulation training using focus group discussions. A total of 19 participants engaged in disaster scenarios with standardized patients, using a PFA app for guidance. Workers valued the practical educational approach, felt increased self-efficacy to support survivors, and identified areas for enhancing simulations and app tools to optimize effectiveness.

### Implementation Outcomes

Implementation outcomes refer to the effects of an intervention or program implementation on various aspects of the implementation process, such as the fidelity of implementation, acceptability, adoption, feasibility, and maintainability. In the papers reviewed, feasibility was assessed using enrollment, program participation, and retention. Acceptability was measured using how well participants liked the app using a rating scale, how much of the app program was completed, the biggest barriers, and whether the app would be recommended to others. Data on characteristics of app use (engagement) were measured using the total number of log ins, average log ins per program completer, platform used (iOS, Android, or web-based), day of week of use (weekday vs weekend), and time of day of use (in 4-hour blocks) [30,31].

The Headspace app was reported to be cost-effective to implement and easy to use [31]. For engagement, only 14% (43/318) of the enrolled women used the app. The level of engagement with the app was high, with 72% (31/43) of participants completing some or all the sessions. Retention was also high with 74% (32/43) of the participants completing the follow-up survey. Lack of time was cited as the main barrier to using the app for 37% (16/43) of users and 49% (94/193) of nonusers. The majority of the users (32/43, 74%) reported high levels of satisfaction with the app. Acceptability was also high, with most participants (32/43, 74%) reporting that they liked the app and 86% (37/43) reporting that they would recommend it to others. Characteristics of app use showed that of the 1530 log ins, most participants (n=1191, 78%) used the iOS platform, mainly on weekdays (n=1147, 75%) and at different times of day mostly from noon to 4 PM (n=375, 25%).

Sonoma Rises was found to be feasible in terms of engagement and satisfaction among teens with high levels of disaster-related posttraumatic stress symptoms [30]. The self-assessment and data visualization features of the Sonoma Rises app strongly appealed to all the participants, and they were willing to recommend the app to their friends. Self-satisfaction with the mobile app was rated as extremely high (mean 8.50, SD 0.58, on a scale of 0 to 10, with 10 as totally satisfied). The participants agreed or strongly agreed to recommend this intervention to a friend. The participants found the intervention helpful (mean 2, SD 0.82); had the content, functions, and capabilities they needed (mean 3, SD 1.12); and were satisfied with how easy it was to use the app (mean 2, SD 0), on a scale of 1 to 5 with 1 as strongly agree and 5 as strongly disagree. In the qualitative feedback, to make the use of the app better, the participants suggested more notifications to return to the app and the use of the app immediately after a disaster. Implementation outcome was not an objective of the TLS app, hence, none was reported.

### Other Mobile Apps With Potential Use in Disasters

Some mobile apps not meeting the inclusion criteria showed promise for supporting mental health in disasters. PTSD Coach provides tools for managing PTSD symptoms [35]. Though not disaster-specific, its psychoeducation, symptom tracking, and coping strategies could aid survivors. Similarly, COVID Coach was designed to help manage pandemic-related stress and anxiety [36]. These apps are summarized in Table 2.

**Table 2:** Summary of other studies using mobile phone apps for mental health responses.

References	Country of study	Type of natural disaster	Mental health conditions targeted	Name of app (if available)	Intervention (app content)	Conclusion
Kuhn et al [35]	Australia, Canada, Netherlands, Germany, Sweden, and Denmark	None. Designed for general use in PTSD^a^, but could have potential use in disaster response.	PTSD	PTSD Coach	The learn section: psychoeducational information about, professional care, and PTSD and the family. Track symptoms: PTSD checklist and interpretive feedback. Manage symptoms: coping tools for acute distress related to PTSD symptoms. Get support: access to crisis support resources.	PTSD Coach was found to be acceptable and perceived to be helpful for managing PTSD symptoms by veterans and community samples. It also demonstrated improvements in depression symptoms and psychosocial functioning.
Jaworski et al [36]	United States	None. Used in COVID-19 pandemic.	Stress and anxiety	COVID Coach	Tools for coping with stress and challenging situations, psychoeducation, tracking of mental health symptoms, and access to networks and crisis support. Tools for managing symptom adapted for life through the pandemic. Psychoeducational topics about managing COVID-19–related issues.	The use of COVID Coach suggests that mobile apps might have the spread and accessibility needed to act as a useful medium for distributing mental health information and resources to persons enduring COVID-19 pandemic–related stress.

^a^PTSD: posttraumatic stress disorder.

## Discussion

### Principal Findings

This review sought to identify and map the use of mobile apps for the mental health component of natural disaster management. We found only 5 studies meeting the inclusion criteria. The scarcity of published literature in this area suggests that mobile apps have not been extensively used in mental health responses to natural disasters. Academic studies on the public’s use of mobile technologies in disaster management are still nascent [37], but there has been increased interest in developing and deploying digital technology and mobile apps by governments and nonstate actors as part of disaster preparedness and response [38,39]. A recent systematic review found that there is a lack of mental health preparedness in most countries when it comes to disasters [40]. The 5 studies included in our scoping review confirmed this gap and further demonstrated that mobile apps can provide mental health support to disaster-affected individuals and communities. The studies found that the use of mobile apps was associated with improvements in mental health outcomes, such as decreased anxiety and depression symptoms and increased resilience. The reviewed studies also suggest that mobile apps can be effective in delivering psychoeducation and coping skills training to disaster-affected individuals. A 2017 scoping review found that mobile apps have been largely used for communication purposes in disaster management [37]. The scope of use was classified into 5 categories which are not mutually exclusive. These categories are (1) crowdsourcing (organize and collect disaster-related data from the crowd), (2) collaborating platforms (serve as a platform for collaboration during disasters), (3) alerting and information (disseminate authorized information before and during disasters), (4) collating (gather, filter, and analyze data to build situation awareness), and (5) notifying (for users to notify others during disasters) [37].

**Figure 2:**
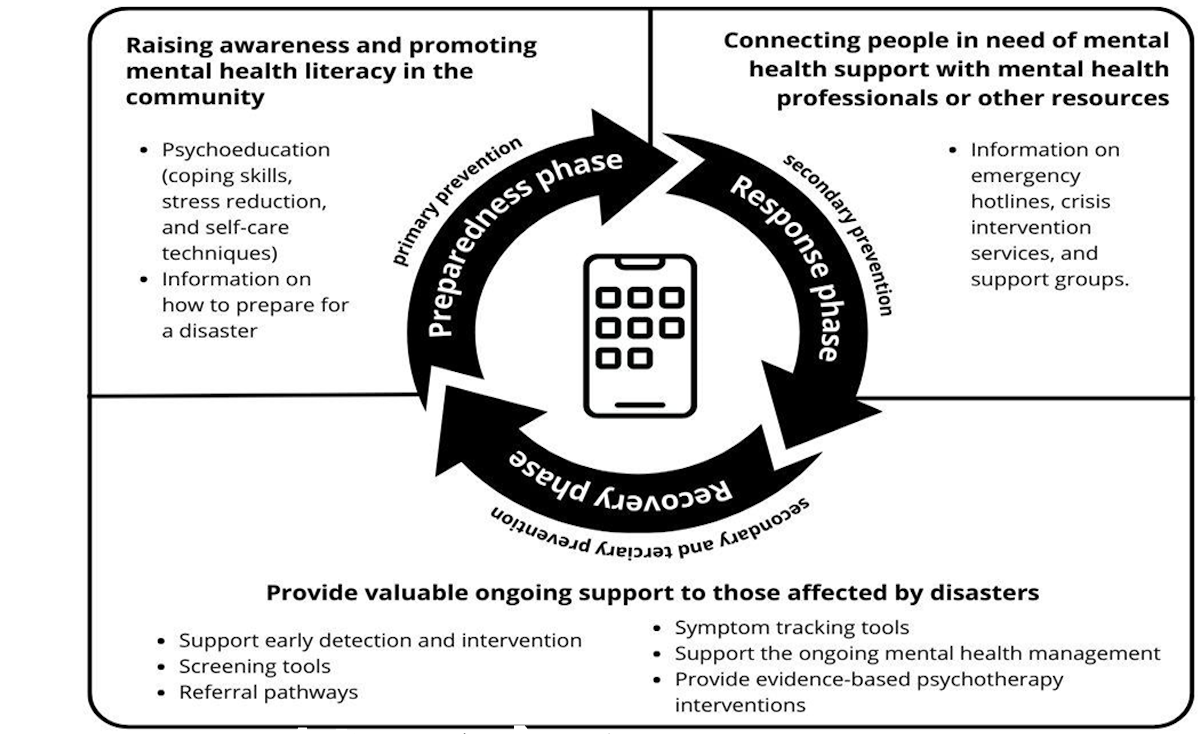
Potential opportunities for mobile apps use in disasters.

Some authors classify disaster response into 3 phases: preparedness, response, and mitigation [41]. The studies included in this review exclusively examined the use of mobile apps during the recovery phase of disaster management. However, none of the studies explored the potential of mobile apps during the preparedness or response phases of disaster management. By addressing this gap, future research could help to provide more comprehensive and effective strategies for the use of mobile apps throughout all phases of disaster management. Examples of potential opportunities are demonstrated in Figure 2.

### Preparedness Phase

Mobile apps can play a critical role as primary prevention interventions by raising awareness and promoting mental health literacy in the community in preparation for natural disasters. These apps can provide information on common mental health problems that may arise during and after disasters and offer tips on staying mentally healthy. For example, apps can include psychoeducation modules on coping skills, stress reduction, and self-care techniques, as well as information on how to prepare for a disaster and what steps to take to protect one’s mental health during and after a disaster. The use and effectiveness of mobile apps in health literacy have been demonstrated in the literature [19], thus providing a foundation for adaptation in disaster management.

### Response Phase

Mobile apps can be used to connect people in need of mental health support with mental health professionals or other resources. For example, apps can provide information on emergency hotlines, crisis intervention services, and support groups. This was demonstrated as effective during the COVID-19 pandemic [42]. Mobile apps can also provide coping strategies and techniques to manage stress and anxiety in response to other natural disasters [34]. In this scoping review, we found that 3 apps had positive benefits in addressing mental health issues among persons exposed to natural disasters.

### Recovery Phase

As part of secondary and tertiary prevention strategies, mobile apps can provide valuable ongoing support to those affected by disasters. For secondary prevention, mobile apps can be designed to support early detection and intervention for mental health problems after a natural disaster. These apps can include screening tools to identify common mental health issues such as anxiety, depression, and PTSD and offer appropriate referral pathways [43]. Additionally, apps can provide symptom-tracking tools to help individuals monitor their mental health over time [43]. For tertiary prevention, mobile apps can support the ongoing management of established mental health problems after a natural disaster. For example, apps can provide evidence-based psychotherapy interventions, such as cognitive-behavioral therapy, to help individuals manage their symptoms [44]. They can also connect individuals with support groups and peer-to-peer networks to provide additional emotional support and help individuals connect with others who have experienced similar challenges. Furthermore, mobile apps can offer self-help tools, such as meditation exercises and mood tracking, to help people cope with the ongoing mental health effects of the disaster. They can also provide information on local mental health services and support groups, helping individuals access the resources they need to manage their mental health.

### General Mental Health Apps Show Promise for Disaster Response

While not specifically designed for disaster contexts, some mobile apps demonstrate strategies to support mental health that could aid disaster survivors. PTSD Coach delivers PTSD psychoeducation, symptom tracking tools, coping skills training, and crisis resource access—elements that could help survivors experiencing common postdisaster issues like trauma or loss [35]. Though it was tailored for veterans and civilians with PTSD, 1 study found it improved users’ depression and functioning. Similarly, COVID Coach offered pandemic-related stress management through symptom tracking, healthy coping recommendations, and crisis line referrals [36]. By leveraging the scalability of mobile apps, COVID Coach reached many struggling during a global crisis. These examples illustrate that apps may provide accessible, far-reaching mediums for disseminating disaster mental health resources—even without disaster-specific tailoring. Research should further explore adapting evidence-based, general mental health apps for disaster contexts or incorporate elements of them into future disaster response tools. With mental health needs magnified during disasters, mobile apps with thoughtful design show promise in expanding access to psychosocial support.

There are several potential limitations when using mobile apps for mental health responses to disasters. One of the main concerns is the accessibility of these apps, as not all members of the affected communities may have access to smartphones or internet connectivity. Furthermore, language and cultural barriers may prevent effective use. Another potential limitation is the quality and accuracy of the information provided. Without proper oversight, some apps may provide misinformation or inaccurate advice, which could exacerbate mental health issues. In addition, privacy concerns around collecting and storing sensitive data must be addressed.

Barriers like lack of mobile devices and internet access can impede adoption, especially in marginalized areas. Apps not designed for low literacy users or that are only available in certain languages could also limit accessibility. Concerns around privacy and security may deter some individuals. However, smartphone ubiquity globally enables use by vulnerable groups. Government agencies and nongovernmental organizations (NGOs) can promote adoption by integrating vetted apps into disaster protocols and funding dissemination. Developing apps with stakeholders and prelaunch user testing also facilitate uptake. Monitoring user feedback allows for ongoing optimization and troubleshooting of barriers. Cultural tailoring to address stigma and use local beliefs further enables implementation success. Finally, limited evidence-based research into app effectiveness highlights the need for more rigorous evaluation and testing of mobile apps for disaster mental health response.

This scoping review has certain methodological limitations that should be considered while interpreting its results. First, the search was restricted to 6 electronic databases and only English-language papers were considered. We also searched MEDLINE and not PubMed, and these may have led to the omission of some relevant studies. Second, the study focused on mobile phone apps for mental health response to disasters, disregarding other types of technology that could also be used in disaster management such as telehealth, SMS text messaging, and emails. Moreover, since the study included only 5 papers, it may not offer a comprehensive overview of the use of mobile phone apps in disaster response strategies. There is the possibility of the existence of apps not yet published in academic literature. Fourth, the nonuse of a control group in the design of the studies makes it difficult to determine whether the observed effects were entirely due to the use of the apps or other characteristics of the participants that predisposed them to use the apps. Fifth, the small sample sizes for the studies mean they require caution with generalization. Despite these limitations, the review provides valuable insights into the use of mobile apps in disaster response and serves as a useful resource for developing contextually appropriate mobile apps for disaster management. Last, our study focused on natural disasters, further research should examine the role of apps in supporting mental health in conflict and complex emergencies such as wars, outbreaks of violence, and complex political conflict situations [45].

### Conclusions

This scoping review found that mobile apps have not been extensively used in mental health responses to natural disasters, with only 5 studies meeting the inclusion criteria. However, the studies included in this review demonstrate that mobile apps can be useful in providing mental health support to disaster-affected individuals, as well as equip disaster responders. There is a critical gap identified in this study, as none of the studies investigated the use of mobile apps for potential victims in the preparedness or response phases of disaster management. We, therefore, recommend that mobile apps be integrated into the various phases of disaster management as part of mental health response. Additionally, it is important to ensure that these apps are accessible to all members of the community, taking into account cultural, linguistic, and other factors that may impact their effectiveness. Mobile apps have great potential to provide valuable ongoing support to those affected by disasters, and they can be a valuable resource in disaster management, helping people cope with the mental health effects of disasters and connecting with the necessary support services.

The findings from this scoping review have important implications for policy makers, disaster management professionals, and mental health practitioners. There is a clear need for policies and protocols that integrate evidence-based mobile apps into mental health disaster planning and response. Disaster agencies should invest in developing, evaluating, and widely disseminating mobile apps specifically designed to mitigate psychological trauma before, during, and after catastrophic events. Mental health professionals can incorporate vetted mobile apps into their standard of care for at-risk disaster survivors. Going forward, a collaborative approach across these groups will be essential to leverage mobile technology in building community resilience and addressing the rising mental health burdens in an era defined by climate change–fueled natural disasters.

## Appendix

Multimedia Appendix 1 The PRISMA-SCR checklist. PRISMA-SCR: Preferred Reporting Items for Systematic Reviews and Meta-Analyses extension for Scoping Reviews.

Multimedia Appendix 2 Detailed search strategy.

## Abbreviations

BFS: Behaviour and Feelings Survey
CESD-10: Center for Epidemiologic Studies Depression Scale-10
CPSS-5: Child PTSD Symptom Scale for Diagnostic and Statistical Manual of Mental Disorders, Fifth Edition
*DSM-5*: *Diagnostic and Statistical Manual of Mental Disorders, Fifth Edition*
EEG: electroencephalogram
EP: explanatory power
GAD-7: Generalized Anxiety Disorder-7
HIPAA: Health Insurance Portability and Accountability Act
ISI: Insomnia—Severity Index
MAAS: Mindful Attention Awareness Scale
mHealth: mobile health
NGO: Non-Governmental Organization
OR: odds ratio
OSY: Ohio Scale for Youth—Functioning subscale
PFA: Psychological First Aid
PHQ-9: Patient Health Questionnaire-9
PRISMA-ScR: Preferred Reporting Items for Systematic Reviews and Meta-Analyses extension for Scoping Reviews
PSQI: Pittsburgh Sleep Quality Index
PSS-4: Perceived Stress Scale, 4-item version
PTSD: posttraumatic stress disorder
SAMHSA: Substance Abuse and Mental Health Services Administration
SEI: Student Engagement Instrument
TLS: Training for Life Skills
WEMWBS: Warwick-Edinburgh Mental Well-being Scale
WSSS: Wills’ Social Support Scale
