# Supplementary material for: Mobile Apps to Support Mental Health Response in Natural Disasters: Scoping Review
Source: J Med Internet Res. 2024 Apr 17;26:e49929. doi: 10.2196/49929 (PMC11063879; doi:10.2196/49929)
Supplement: Multimedia Appendix 2 [file jmir_v26i1e49929_app2.docx]

**Search Strategies**

**MEDLINE**

[Ovid MEDLINE(R) ALL 1946 to June 29, 2022]

1. (mhealth or m-health).mp.

2. mobile health.mp.

3. exp Cell Phone/ or exp Mobile Applications/

4. exp Computers, Handheld/

5. mobile device*.mp.

6. ((portable or mobile or smart or tablet or cell*) adj5 (app or apps or application* or phone* or telephone*)).mp.

7. (android or iphone* or i-phone*).mp.

8. or/1-7

9. public health emergenc*.mp.

10. disaster*.mp. or exp Disasters/

11. emergency preparedness.mp.

12. emergency management.mp.

13. risk communication*.mp.

14. emergency communication*.mp.

15. catastrophe*.mp.

16. (flood* or earthquake* or hurricane* or typhoon* or fire or fires or wildfire* or bushfire* or storm or storms or tornado* or drought or cyclone* or blizzard* or tsunami*).mp.

17. or/9-16

18. exp Mental Health/

19. (wellness or wellbeing or well-being).mp.

20. exp Resilience, Psychological/

21. (psychologic* or psychiatric*).mp.

22. (stress* or anxiet* or depression* or emotional).mp.

23. (posttraumatic or post-traumatic or PTSD).mp.

24. ((mental* or psychologic* or psychiatric) adj3 (health* or well* or ill* or disease* or disorder*)).ti,ab.

25. exp Mental Disorders/ or exp Anxiety Disorders/ or exp Mood Disorders/ or exp Neurotic Disorders/ or exp Psychotic Disorders/ or exp Stress, Psychological/ or exp Substance-Related Disorders/

26. (addiction* or "substance use" or substance abuse or alcohol* or "drug use" or drug abuse or drug misuse).ti,ab,kf.

27. or/18-26

28. 8 and 17 and 27

29. limit 28 to english language

**Embase**

[Ovid Embase 1974 to 2022 June 29]

1. (mhealth or m-health).mp.

2. mobile health.mp.

3. exp mobile phone/ or exp mobile applications/

4. exp personal digital assistant/

5. mobile device*.mp.

6. ((portable or mobile or smart or tablet or cell*) adj5 (app or apps or application* or phone* or telephone*)).mp.

7. (android or iphone* or i-phone*).mp.

8. or/1-7

9. public health emergenc*.mp.

10. disaster*.mp. or exp disaster/

11. emergency preparedness.mp.

12. emergency management.mp.

13. risk communication*.mp.

14. emergency communication*.mp.

15. catastrophe*.mp.

16. (flood* or earthquake* or hurricane* or typhoon* or fire or fires or wildfire* or bushfire* or storm or storms or tornado* or drought or cyclone* or blizzard* or tsunami*).mp.

17. or/9-16

18. exp mental health/

19. (wellness or wellbeing or well-being).mp.

20. exp psychological resilience/

21. (psychologic* or psychiatric*).mp.

22. (stress* or anxiet* or depression* or emotional).mp.

23. (posttraumatic or post-traumatic or PTSD).mp.

24. ((mental* or psychologic* or psychiatric) adj3 (health* or well* or ill* or disease* or disorder*)).ti,ab.

25. exp *mental disease/ or exp addiction/ or exp *anxiety disorder/ or exp behavior disorder/ or exp emotional disorder/ or exp *mood disorder/ or exp neurosis/ or exp psychosis/ or exp psychological well-being/ or exp drug dependence/

26. (addiction* or "substance use" or substance abuse or alcohol* or "drug use" or drug abuse or drug misuse).ti,ab,kw.

27. or/18-26

28. 8 and 17 and 27

29. limit 28 to english language

**PsycINFO**

[APA PsycInfo 1806 to June Week 3 2022]

1. (mhealth or m-health).mp.

2. mobile health.mp.

3. exp Mobile Phones/ or exp Mobile Applications/

4. mobile device*.mp.

5. ((portable or mobile or smart or tablet or cell*) adj5 (app or apps or application* or phone* or telephone*)).mp.

6. (android or iphone* or i-phone*).mp.

7. or/1-6

8. public health emergenc*.mp.

9. disaster*.mp. or exp Disasters/

10. emergency preparedness.mp.

11. emergency management.mp.

12. risk communication*.mp.

13. emergency communication*.mp.

14. catastrophe*.mp.

15. (flood* or earthquake* or hurricane* or typhoon* or fire or fires or wildfire* or bushfire* or storm or storms or tornado* or drought or cyclone* or blizzard* or tsunami*).mp.

16. or/8-15

17. exp Mental Health/

18. (wellness or wellbeing or well-being).mp.

19. exp "Resilience (Psychological)"/

20. (psychologic* or psychiatric*).mp.

21. (stress* or anxiet* or depression* or emotional).mp.

22. (posttraumatic or post-traumatic or PTSD).mp.

23. ((mental* or psychologic* or psychiatric) adj3 (health* or well* or ill* or disease* or disorder*)).ti,ab.

24. exp Mental Disorders/ or exp Anxiety Disorders/ or exp Affective Disorders/ or exp Neurosis/ or exp Psychosis/ or exp Psychological Stress/ or exp "Substance Use Disorder"/

25. (addiction* or "substance use" or substance abuse or alcohol* or "drug use" or drug abuse or drug misuse).ti,ab.

26. or/17-25

27. 7 and 16 and 26

28. limit 27 to english language

**CINAHL**

S1 mhealth or m-health

S2 "mobile health"

S3 (MH "Cellular Phone+")

S4 (MH "Mobile Applications")

S5 (MH "Computers, Hand-Held+")

S6 "mobile device*"

S7 ((portable or mobile or smart or tablet or cell*) N5 (app or apps or application* or phone* or telephone*))

S8 android or iphone* or i-phone*

S9 S1 OR S2 OR S3 OR S4 OR S5 OR S6 OR S7 OR S8

S10 public health emergenc*

S11 (MH "Disasters+") OR "disaster*"

S12 "emergency preparedness"

S13 "emergency management"

S14 "risk communication*"

S15 "emergency communication*"

S16 catastrophe*

S17 flood* or earthquake* or hurricane* or typhoon* or fire or fires or wildfire* or bushfire* or storm or storms or tornado* or drought or cyclone* or blizzard* or tsunami*

S18 S10 OR S11 OR S12 OR S13 OR S14 OR S15 OR S16 OR S17

S19 (MH "Mental Health")

S20 wellness or wellbeing or well-being

S21 (MH "Stress, Psychological+")

S22 psychologic* or psychiatric*

S23 stress* or anxiet* or depression* or emotional

S24 posttraumatic or post-traumatic or PTSD

S25 TI ( ((mental* or psychologic* or psychiatric) N3 (health* or well* or ill* or disease* or disorder*)) ) OR AB ( ((mental* or psychologic* or psychiatric) N3 (health* or well* or ill* or disease* or disorder*)) )

S26 (MH "Mental Disorders+")

S27 (MH "Anxiety Disorders+")

S28 (MH "Affective Disorders+")

S29 (MH "Neurotic Disorders+")

S30 (MH "Psychotic Disorders+")

S31 (MH "Substance Use Disorders+")

S32 TI ( (addiction* or "substance use" or substance abuse or alcohol* or "drug use" or drug abuse or drug misuse) ) OR ( (addiction* or "substance use" or substance abuse or alcohol* or "drug use" or drug abuse or drug misuse) )

S33 S19 OR S20 OR S21 OR S22 OR S23 OR S24 OR S25 OR S26 OR S27 OR S28 OR S29 OR S30 OR S31 OR S32

S34 S9 AND S18 AND S33

S35 S9 AND S18 AND S33 [Limiters: English language]

**Scopus**

( TITLE-ABS-KEY ( ( mhealth  OR  m-health  OR  "mobile health"  OR  "mobile device*"  OR  ( ( portable  OR  mobile  OR  smart  OR  tablet  OR  cell* )  W/5  ( app  OR  apps  OR  application*  OR  phone*  OR  telephone* ) )  OR  android  OR  iphone*  OR  i-phone* ) )  AND  TITLE-ABS-KEY ( ( "public health emergenc*"  OR  disaster*  OR  "emergency preparedness"  OR  "emergency management"  OR  "risk communication*"  OR  "emergency communication*"  OR  catastrophe*  OR  flood*  OR  earthquake*  OR  hurricane*  OR  typhoon*  OR  fire  OR  fires  OR  wildfire*  OR  bushfire*  OR  storm  OR  storms  OR  tornado*  OR  drought  OR  cyclone*  OR  blizzard*  OR  tsunami* ) )  AND  TITLE-ABS-KEY ( ( wellness  OR  wellbeing  OR  well-being  OR  psychologic*  OR  psychiatric*  OR  stress*  OR  anxiet*  OR  depression*  OR  emotional  OR  posttraumatic  OR  post-traumatic  OR  ptsd  OR  ( ( mental*  OR  psychologic*  OR  psychiatric )  W/3  ( health*  OR  well*  OR  ill*  OR  disease*  OR  disorder* ) )  OR  addiction*  OR  "substance use"  OR  "substance abuse"  OR  alcohol*  OR  "drug use"  OR  "drug abuse"  OR  "drug misuse" ) ) )  AND  ( LIMIT-TO ( LANGUAGE ,  "English" ) )

**Web of Science Core Collection**

TS=(mhealth or m-health or "mobile health" or "mobile device*" or ((portable or mobile or smart or tablet or cell*) NEAR/5 (app or apps or application* or phone* or telephone*)) or android or iphone* or i-phone*) and TS=("public health emergenc*" or disaster* or "emergency preparedness" or "emergency management" or "risk communication*" or "emergency communication*" or catastrophe* or flood* or earthquake* or hurricane* or typhoon* or fire or fires or wildfire* or bushfire* or storm or storms or tornado* or drought or cyclone* or blizzard* or tsunami*) and TS=(wellness or wellbeing or well-being or psychologic* or psychiatric* or stress* or anxiet* or depression* or emotional or posttraumatic or post-traumatic or PTSD or ((mental* or psychologic* or psychiatric) NEAR/3 (health* or well* or ill* or disease* or disorder*)) or addiction* or "substance use" or "substance abuse" or alcohol* or "drug use" or "drug abuse" or "drug misuse")

Refined by - Languages: English
